# Categorizing update mechanisms for graph-structured metapopulations

**DOI:** 10.1098/rsif.2022.0769

**Published:** 2023-03-15

**Authors:** Sedigheh Yagoobi, Nikhil Sharma, Arne Traulsen

**Affiliations:** Department of Evolutionary Theory, Max Planck Institute for Evolutionary Biology, August-Thienemann Strasse 2, Plön 24306, Germany

**Keywords:** evolutionary graph theory, graph-structured metapopulation, network-structured metapopulation, update mechanism

## Abstract

The structure of a population strongly influences its evolutionary dynamics. In various settings ranging from biology to social systems, individuals tend to interact more often with those present in their proximity and rarely with those far away. A common approach to model the structure of a population is evolutionary graph theory. In this framework, each graph node is occupied by a reproducing individual. The links connect these individuals to their neighbours. The offspring can be placed on neighbouring nodes, replacing the neighbours—or the progeny of its neighbours can replace a node during the course of ongoing evolutionary dynamics. Extending this theory by replacing single individuals with subpopulations at nodes yields a graph-structured metapopulation. The dynamics between the different local subpopulations is set by an update mechanism. There are many such update mechanisms. Here, we classify update mechanisms for structured metapopulations, which allows to find commonalities between past work and illustrate directions for further research and current gaps of investigation.

## 1. Introduction

The spatial structure of a population has a considerable impact on the evolutionary dynamics of a population. One of the most popular theories for studying the effect of underlying structure on the evolution of a population is evolutionary graph theory [1], where the nodes of a graph represent individuals, and links indicate individual’s neighbours. A link determines where an individual can place their offspring. An update mechanism describes which individuals produce offspring and how this offspring is placed. Here, we focus on fixed structures, but the framework can be extended to the case where a spatial structure itself varies over time [2–4].

An extension of evolutionary graph theory is given by graph-structured metapopulations in which the nodes indicate subpopulations, and the links indicate the migration between subpopulations [5–8]. Evolutionary dynamics in subdivided populations has been studied by researchers for a long time [9]. Models investigating the evolution of frequencies of different individuals in a subdivided population are also known as ‘island models’ [10]. A simplified version of these models considers individuals migrating from every island (subpopulation) to all the other islands with the assumption of a constant migration rate. In the language of evolutionary graph theory this is equivalent to the migration dynamics on a complete graph of subpopulations. To make this model more realistic, the stepping stone model was introduced where the migration rate depends on the distance between islands [11]. For a long time, the studies were mainly focused on the fully connected metapopulation. Patwa & Wahl [12] gives a good overview of the earlier work done on fixation probabilities for metapopulations. More recently, structures other than the complete graph are also being investigated. As an example, the star graph-structured metapopulation, where nodes are connected to each other via a central node, is studied in Constable & McKane [5], Yagoobi & Traulsen [6] and Marrec *et al.* [7]. Depending on the system, different update mechanisms have been applied to describe the dynamics. In general, evolutionary dynamics are not robust to the choice of update mechanisms [13–16]. An important factor that changes the system’s dynamics is how selection acts.

Depending on the update mechanism, selection can be global or local. This can affect the evolutionary dynamics dramatically. The two most important events that govern the dynamics of the population are birth and death. The order in which birth and death occur, which often determines if selection is local or global, has a high impact on the fate of the population [17]. We categorize different update mechanisms for the evolution of structured metapopulations to facilitate future work. First, we recall the update mechanisms on graphs of individuals, and then we generalize them to graphs of subpopulations.

## 2. Update mechanisms for graphs of individuals

In graphs of individuals, three main events influence the evolution of a population: birth, mutation and death. In the long run, a mutation–selection balance is reached [8,18–21]. Typically, the focus is on the low mutation regime in which the system reaches fixation or extinction of the mutant before the next mutation occurs. In that case, the population is typically homogeneous, and mutations reach fixation one after another. In the low mutation regime, the two quantities of most interest are the fixation probability and the average time to fixation.

If the fixation probability for advantageous mutants on a graph is higher than the fixation probability of the complete graph (and vice versa for disadvantageous mutations), the graph is called an amplifier of selection. On the other hand, if the fixation probability for advantageous mutants on a graph is less than the fixation probability on the complete graph (and vice versa for disadvantageous mutations), this graph is called a suppressor of selection. These notions have been first introduced in [1].

The order of birth and death events and how selection acts upon them can substantially influence the population’s fate. We use the following scheme to differentiate between the update mechanisms. For birth, we use **b** if birth is random, i.e. a birth-giving individual is chosen uniformly at random, and **B** if the birth-giving individual is chosen with probability proportional to its selection parameter for birth. Similarly, we represent the death event by **d** if the individual dies uniformly at random and by **D** if the individual is selected for death with a probability proportional to its selection parameter for death.

Accordingly, the eight possible update mechanisms are **BD**, **Bd**, **bD**, **bd**, **DB**, **Db**, **dB** and **db**, where the order shows which event is first (see table 1). In all these update mechanisms, the first event is global, meaning that the individual is selected from the whole population. By contrast, the second event is local because the individual is selected only from the neighbourhood of the first individual, which is a subset of the population.

**Table 1.** Update mechanisms in graphs of individuals. In these update mechanisms, birth and death change the state of the population. The first event is global and the second event is local.

update mechanism	comment	references
**BD**	—	[17,22,23]
**Bd**	—	[1,17,22,24–40]
**bD**	—	[17,22,27,28,36]
**bd**	equivalent to a completely neutral model	[17,36,39–41]
**DB**	—	[17,22]
**Db**	biased voter model	[17,22,26–28,32,36,42–44]
**dB**	biased voter model	[16,17,22,27–31,36,45–47]
**db**	equivalent to a completely neutral model	[17,41]

As an example, applying the update mechanism **Bd** in a well-mixed population of size *N* consisting of two types of individuals, *N* − *n* wild-types and *n* mutants, with mutants having a relative selection parameter for birth *r* with respect to wild-types. Let us consider that the number of mutants in the population is increased by one: one mutant is selected for reproduction (global event). Then, from the neighbours of the mutant, one wild-type is selected for death (local event). The offspring fills the empty spot of the dead individual. The probability of increasing the number of mutants by one is then

2.1 $$T_{\mathrm{Bd}}^{n+}=\underset{\mathrm{birth}\;\mathrm{of}\;\mathrm{mutant}}{\underbrace{\frac{rn}{rn+N-n}}}\underset{\mathrm{death}\;\mathrm{of}\;\mathrm{wild-type}}{\underbrace{\frac{N-n}{N-1}}}.$$

Similarly, the probability of decreasing the number of mutants by one is

2.2 $$T_{\mathrm{Bd}}^{n-}=\underset{\mathrm{birth}\;\mathrm{of}\;\mathrm{wild-type}}{\underbrace{\frac{N-n}{rn+N-n}}}\underset{\mathrm{death}\;\mathrm{of}\;\mathrm{mutant}}{\underbrace{\frac{n}{N-1}}}.$$

Note that $T_{\mathrm{Bd}}^{n-}/T_{\mathrm{Bd}}^{n+}=1/r$. Using the recursive relation for the fixation probability [24,48], the fixation probability starting from *n* mutants is

2.3 $$\phi^{\left(n\right)}=\frac{1+\sum_{i=1}^{n-1}\prod_{j=1}^{i}\frac{T^{j+}}{T^{j-}}}{1+\sum_{i=1}^{N-1}\prod_{j=1}^{i}\frac{T^{j+}}{T^{j-}}}=\frac{1-\frac{1}{r^{n}}}{1-\frac{1}{r^{N}}}.$$

The update rule **Bd** has been vastly explored in both structured and well-mixed populations [1,22,25,42,45,49,50]. For small populations, under the update mechanism **Bd**, most undirected unweighted structures are amplifiers of selection, and only a small fraction of random structures suppress selection [15]. In general, the fixation probabilities for mutants are computed using numerical approaches [51,52]. Only for highly symmetric graphs like the star graph or the complete bipartite graph, can the fixation probability be computed analytically [25,53]. Under the **Bd** updating scheme, the star graph is an amplifier of selection for all population sizes [1]. In [54], a few small-sized undirected, unweighted suppressors of selection have been studied. In larger populations, in the weak selection regime, a lot of graphs have been shown to suppress selection [55,56]. Furthermore, under **Bd** updating, regular structures have the same fixation probability as the complete graph regardless of the mutant’s fitness. To be specific, a graph where the total incoming weight to all the nodes are equal, then according to the ‘isothermal theorem’ [1,24] the graph has the same fixation probability as the complete graph.

Many studies are also dedicated to **dB** updating [15–17,22,45–47,49,57,58]. Contrary to **Bd**, under **dB** updating only a small fraction of undirected random graphs amplify selection while the majority of graphs suppresses selection [15]. The star graph is a suppressor of selection under the **dB** update mechanism [13,15]. Another popular update mechanism is **Db** which is equivalent to the voter model in statistical physics [26,42,50,59], but which is also used in biology [43]. In [22], it is shown that the evolutionary dynamics on a lattice under update mechanisms **Bd** and **Db** are equivalent when the selection parameter for death in the update mechanism **Db** equals the inverse of selection parameter for birth in the update mechanism **Bd**. However, it is illustrated that the dynamics on this lattice under update mechanisms **dB** and **bD** are fundamentally different.

**Figure 1.**
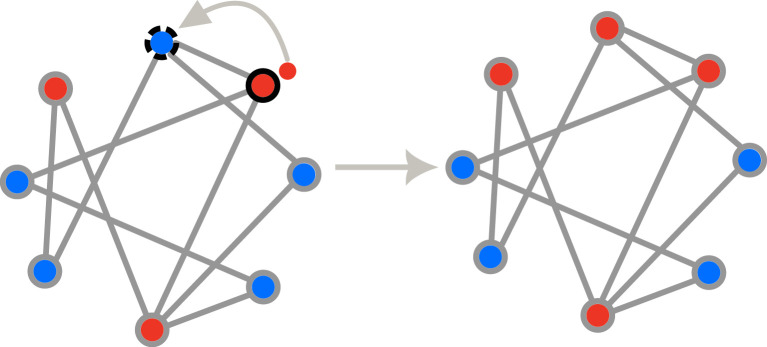
Different update schemes for graph of individuals. We consider an arbitrary population structure of size eight with five wild-type individuals (blue) and three mutant individuals (red). Neighbours are connected via links. Individual marked with solid black circle represents the birth giving parent, whereas, the individual marked with black dashed circle is the one chosen for death. The population size remains constant throughout the dynamics with offspring replacing dead individuals. Assuming that the selection parameter for birth of a mutant individual is *r* = 2 (1 for the wild-type) and that the selection parameter for death of the mutant is *t* = 1/2 (1 for the wild-type), the probabilities that the transition shown in the figure takes place are different for the different update mechanisms shown in table 1. For example, in the case of **BD**, the probability to choose this particular mutant individual for birth is $\frac{2}{3\cdot 2+5\cdot 1}=\frac{2}{11}$. The probability to choose this particular wild-type neighbour for death is $\frac{1}{1\cdot 1/2+2\cdot 1}=\frac{2}{5}$, which leads to a probability $\frac{4}{55}\approx 0.072$ for the event shown. Similarly, we find: **Bd**: $\frac{2}{3\cdot 2+5\cdot 1}\cdot \frac{1}{3}\approx 0.061$. **bD**: $\frac{1}{8}\cdot \frac{1}{1\cdot 1/2+2\cdot 1}=0.05$. **bd**: $\frac{1}{8}\cdot \frac{1}{3}\approx 0.042$. **DB**: $\frac{1}{3\cdot 1/2+5\cdot 1}\cdot \frac{2}{1\cdot 2+2\cdot 1}\approx 0.077$. **Db**: $\frac{1}{3\cdot 1/2+5\cdot 1}\cdot \frac{1}{3}\approx 0.51$. **dB**: $\frac{1}{8}\cdot \frac{2}{1\cdot 2+2\cdot 1}\approx 0.062$. **db**: $\frac{1}{8}\cdot \frac{1}{3}\approx 0.042$.

Among the eight update mechanisms for graphs of individuals, **bd** and **db** are identical, describing a system where natural selection has no role in the evolution of the population [17,41]. For the update mechanisms **DB** and **BD**, selection acts both on death and birth [17,22,23], but they are not equivalent in general. Intuitively, one may expect that the fixation probability of an advantageous mutant is higher in the presence of update mechanisms **DB** and **BD** than in the other update mechanisms—but this is not always the case. The general transition probabilities for these eight update mechanisms are given in appendix A. In figure 1, the transition probabilities for a specific example are given for the eight update mechanisms.

In addition to the above update mechanisms, there are other update mechanisms in which, instead of two individuals (one for birth, one for death), one edge is selected [27,42,44]. Then an individual dies at one end, the other gives birth, and the offspring fills the neighbouring empty spot. In this update mechanism, selection can act on death and/or birth events. Here, we will not consider this kind of update mechanism.

**Figure 2.**
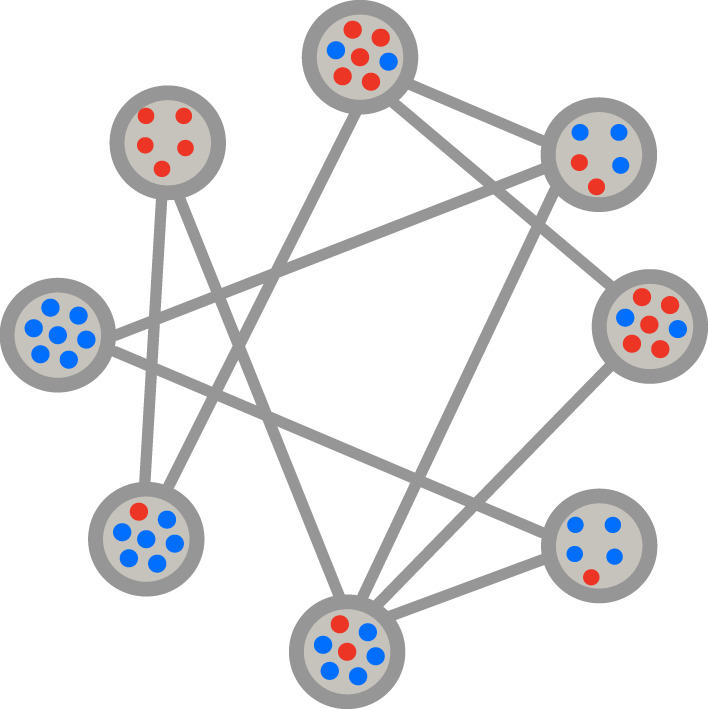
Graph of subpopulations. Each node in the graph includes a well-mixed subpopulation and each link indicates migration between two subpopulations.

For more information about the comparison of different update mechanisms on various graphs with different features, we refer to the following references: in [44], the authors ask when the fixation probability in an evolutionary graph equals the fixation probability in a Moran process. A Moran process [60] is equivalent to the update mechanism **Bd** in a complete graph. In [28], the authors investigate the evolutionary game dynamics on the star graph in the presence of different update mechanisms. They show that the evolutionary dynamics of heterogeneous graphs is not robust under the choice of update mechanism. In [27], the effect of the directionality of a graph on its evolutionary dynamics for different update mechanisms is investigated. It has been shown that regardless of the update mechanism, the directionality always suppresses selection. In this manuscript, we extend the above update mechanisms to graphs of subpopulations where each node comprises a well-mixed subpopulation. The links indicate the migration between the patches (see figure 2).

## 3. Categorizing update mechanisms for graphs of subpopulations

In most of the potential applications of evolutionary graph theory, both in biology and in social dynamics, each node represents a population rather than an individual: individuals tend to interact locally in subpopulations, with some rare interactions with other groups of individuals. This is because the populations are segregated for various reasons, and they are geographically distant. Individuals in the same geographical area compete over resources or provide common goods. However, there is an occasional migration to and from other geographical areas. In [6], it has been shown that for the **Bd** update mechanism, some results of evolutionary graph theory do not carry over into graphs of subpopulations. For example, a star-structured metapopulation does not always amplify selection—in some migration regimes, it suppresses selection. It will be interesting to see if this is the case for other update mechanisms.

**Figure 3.**
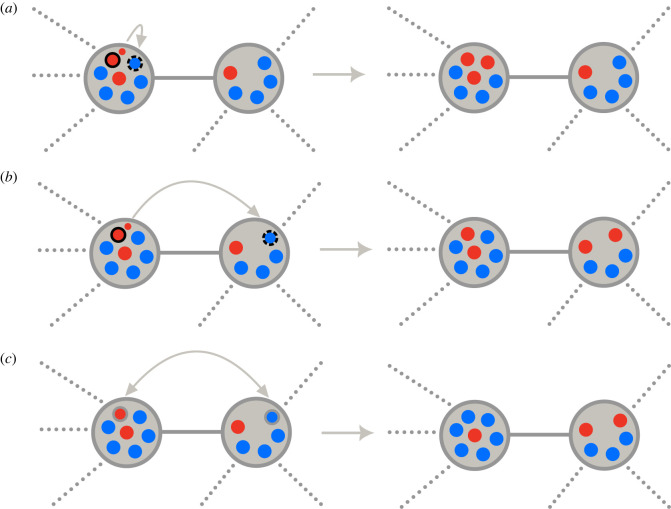
Update mechanisms in a graph-structured metapopulation. The population consists of two types of individuals, wild-types (blue) and mutants (red). The individual marked with the solid black circle gives birth and the individual marked with black dashed circle is selected for death. (*a*) Birth–death or death–birth in one patch without migration: this includes birth–death or death–birth in a coupled update mechanism without migration as well as an uncoupled update mechanism in which both death and birth happen in the same subpopulation. (*b*) A coupled update mechanism with migration: if birth is coupled with migration after each birth the newborn migrates to an adjacent patch and replaces one of its individual. If death is coupled to migration, a death in one patch is followed by birth of an individual in one of the adjacent patches where newborn occupies the place of dead individual. (*c*) Migration in an uncoupled update mechanism: migration happens independently from birth or death and it only exchanges the position of two individuals from different patches. This process is completely random.

In a model with fixed local population sizes, birth and death either happen in the same subpopulation, or the first event can be followed by an individual’s migration to or from another subpopulation. Having this in mind, we can categorize update mechanisms into two groups:

— a set of update rules where there is always the possibility that the first event (birth or death) is accompanied by migration (figure 3*a*,*b*); and
— a set of updates where birth and death events are always in the same subpopulation and migration happens independently from birth and death, (see figure 3*a*,*c*).

We refer to the former category as update mechanisms with coupled migration and the latter as update mechanisms with uncoupled migration. The order of events (birth and death) in each of these classes and how selection acts upon them might affect the dynamics considerably. In both categories of update mechanisms, selection for the first event can act on both patch and individual levels, i.e. one first selects a patch and then an individual from the chosen patch. Selection on the second event can act both on the patch and individual levels in the update mechanisms with coupled migration. However, in the update mechanisms with uncoupled migration, selection in the second event always acts on the individual level since the second event must happen in the same patch as the first event.

We code the update mechanisms as follows: the first letter stands for migration to show if it is coupled (**M**) or uncoupled (**m**). The order of letters, except for the letter for migration, indicates the order of events. In addition, if selection is associated with selection parameters, we assign a capital letter and, otherwise, a lower-case letter. For uncoupled migration we need only three letters (the patch of the second event is fixed), for coupled migration we need four letters (we can select the patch and the individual for the first and for the second event).

### 3.1. Update mechanisms with coupled migration

In this class of update mechanisms, the first event is directly coupled with migration. The first event can be birth or death. If the birth occurs first, one of the patches is selected randomly proportional to the size of the patch (**b**) or randomly proportional to the sum of selection parameters of that patch for birth (**B**). Next, an individual from the patch is selected uniformly at random (**b**) or randomly proportional to its selection parameter for birth (**B**) to produce an identical offspring. The offspring can either stay and substitute one of the individuals in its patch (figure 3*a*) or migrate to one of the neighbouring patches and replace one of the individuals there (figure 3*b*).

If the offspring stays in its own patch, one of the individuals is chosen for death uniformly at random (**d**) or proportional to its selection parameter for death (**D**) (a common choice is the inverse of the selection parameter for birth). If the offspring migrates to a neighbouring patch, an individual in an adjacent patch is selected in two stages. First, among the neighbouring patches, one patch is selected randomly proportional to the size of the patch (**d**) or randomly proportional to the collective selection parameter for death of the patch (**D**). Finally, one of its individuals is selected for death uniformly at random (**d**) or randomly proportional to its selection parameter for death (**D**).

As an example, consider the update mechanism **MbBDd**. In this update mechanism,

(i) First, a patch is selected uniformly at random.
(ii) Then, from the random patch, one individual is selected with probability proportional to its selection parameter for birth to produce an offspring.
(iii) Next, with a certain probability, the offspring will migrate to one of the adjacent patches or remain in its innate patch. In the former case, one of the neighbouring patches is selected uniformly at random as a function of its collective selection parameter for death.
(iv) Finally, in the selected patch, which can be either an adjacent patch or the innate patch, one individual dies uniformly at random, and the offspring fills its empty spot.

In general, selection can be uniformly at random or proportional to a selection parameter in each step.

Based on such procedures, there are 16 different update mechanisms, with birth being the first event (see table 2). Similarly, if the first event is death, there are 16 different update mechanisms (see table 3). So far, only a few of these mechanisms have been studied in detail. For example, **MBBdd** is adopted in [5,6,61]. Not all of the observations made in graphs of individuals with the **Bd** update rule carry over to a graph of subpopulations when the update rule is **MBBdd** [6,61]. In fact, in the graph of subpopulations, the dynamics and, in particular, the fate of advantageous mutants are highly dependent on the pattern of migration, local population size and the graph structure itself. Also, applying **MddBB** in the graph of subpopulations reduces the chance of advantageous mutants compared with the equivalent well-mixed population with the update mechanism **dB** [61]. Furthermore, employing **MddBB** in the star of islands in which many subpopulations are connected only via a central subpopulation, it is shown that it is the relative size of the local population in the leaves and the centre that determines whether the star of islands is an amplifier, reducer or transient amplifier of selection [16]. In general, in this class of update mechanisms, intuition suggests that the more selection is associated with the selection parameters, the more likely a beneficial mutant will spread through the population. However, in appendix D.1, we see that this is not true for the metastar in the low migration rate regime when birth is the first event.

**Table 2.** Birth–death processes with migration coupled to reproduction. In all these update mechanisms in graph-structured metapopulations, the individual producing offspring is identified first and the individual to be removed afterwards. In both steps, we can select for the patch and for the individual separately, leading to 16 such update mechanisms. As an example, the evolutionary dynamics on the metastar under this category of update mechanisms is investigated in appendix D.

update mechanism	comments	references
**MBBDD**	equivalent to **BD** in a graph of individuals	—
**MBBDd**	—	—
**MBBdD**	—	—
**MBBdd**	equivalent to **Bd** in a graph of individuals	[5,6,61]
**MBbDD**	—	—
**MBbDd**	—	—
**MBbdD**	—	—
**MBbdd**	—	[5]
**MbBDD**	—	—
**MbBDd**	—	—
**MbBdD**	—	—
**MbBdd**	—	—
**MbbDD**	equivalent to **bD** in a graph of individuals	—
**MbbDd**	—	—
**MbbdD**	—	—
**Mbbdd**	equivalent to a completely neutral model	—

**Table 3.** Death–birth processes with migration coupled to death. In all these update mechanisms in graph-structured metapopulations, the individual being removed is identified first and the individual producing offspring afterwards. Again, there are 16 such update mechanisms. As an example, the evolutionary dynamics on the metastar under this category of update mechanisms is investigated in appendix D.

update mechanism	comments	references
**MDDBB**	equivalent to **DB** in a graph of individuals	—
**MDDBb**	—	—
**MDDbB**	—	—
**MDDbb**	equivalent to **Db** in a graph of individuals	—
**MDdBB**	—	—
**MDdBb**	—	—
**MDdbB**	—	—
**MDdbb**	—	—
**MdDBB**	—	—
**MdDBb**	—	—
**MdDbB**	—	—
**MdDbb**	—	—
**MddBB**	equivalent to **dB** in a graph of individuals	[16,61]
**MddBb**	—	—
**MddbB**	—	—
**Mddbb**	equivalent to a completely neutral model	—

### 3.2. Equivalence to weighted graphs of individuals

**Figure 4.**
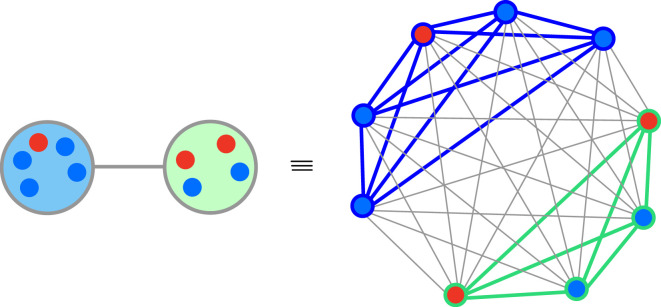
Equivalence of an update mechanism in a graph of subpopulation to an update mechanism in a graph of individuals. In a coupled update mechanism whenever selection on each of the birth and death events both on the patch and individual levels is either uniformly random or randomly proportional to selection parameters, the update mechanism is equivalent to an update mechanism in an associated weighted graph of individuals. In this weighted graph, the weights of the links that connect local individuals are different from the weights of the links that connect individuals in neighbouring subpopulations and depend on the migration probability as well as local population sizes.

In the mechanisms with coupled migration, some update mechanisms reduce to simpler ones in a weighted graph of individuals, where the weights of the links that connect individuals in the same subpopulation are different from the weights of the links that connect individuals in different subpopulations (see figure 4). For instance, for the update rule **MDDBB**, first a patch is selected randomly proportional to its collective selection parameter for death. Afterwards, within the patch, an individual is chosen for death randomly proportional to its selection parameter for death. This is equivalent to selecting one individual from the whole population with a probability proportional to its selection parameter for death. Similarly, selection at birth both at the patch and individual levels is equivalent to selecting an individual for birth proportional to its selection parameter for birth from the whole population (for more details see appendix C).

Hence, **MDDBB** on a graph-structured metapopulation can be treated as a **DB** on the equivalent graph of individuals with weighted links between individuals such that the weights of links that connect the individuals belonging to one patch differ from the ones that connect individuals from different patches. In fact, in the coupled update mechanisms, whenever selection on each of the birth and death events both on the patch level and individual level is either uniformly random or randomly proportional to selection parameters (**BB** or **bb** and **DD** or **dd**), the update mechanism reduces to an update mechanism in an equivalent graph of individuals, as mentioned in tables 2 and 3.

### 3.3. Update mechanisms with uncoupled migration

Migration is said to be uncoupled if birth and death events take place within a single patch (figure 3*a*), and individuals migrate independently such that the population size in each patch remains constant, independent of birth and death (figure 3*c*). In this scenario, we can model migration as follows: with a certain probability, two random individuals from two random connected patches exchange their positions. In this way, the local population size will remain constant.

The first event can be birth or death. If birth happens first, one of the patches is selected uniformly at random (**b**) or randomly proportional to its selection parameter for birth (**B**). From the chosen patch, one individual is selected for birth uniformly at random (**b**) or randomly proportional to its selection parameter for birth (**B**) and produces an identical offspring. Once an individual is selected for birth, one individual is selected for death uniformly at random (**d**) or randomly proportional to its selection parameter for death (**D**) from the same patch. The new offspring replaces the empty spot of the dead individual. Since there is no patch selection for the second event, the second event is indicated by a single letter.

In this category, there are eight different update mechanisms. As an example, let us consider **mbBD**. At each time step, with the migration probability, *λ*, a migration happens, and with the probability, 1 − *λ*, the population gets updated through the birth–death process. The birth–death process is as follows,

(i) First, a patch is selected uniformly at random (**b**).
(ii) From this patch, an individual is selected with probability proportional to its selection parameter for birth to reproduce (**B**).
(iii) After that, one of the individuals from the same patch is chosen randomly with probability proportional to its selection parameter for death to die (**D**) and the offspring will fill the empty spot.

Similarly, when death happens first, there are eight other update mechanisms. In the update mechanisms **mbbd** and **mddb**, where selection is not active in either of the events, the update mechanism is identical to the neutral model, i.e. **bd**.

In many popular models of the population genetics literature, migration is assumed to be independent from birth and death [7,9,11,62–65]. However, most of these studies use the Wright–Fisher model as the local update mechanism and thus are not captured by our metapopulation framework of evolutionary graph theory.

## 4. Discussion

Evolutionary graph theory is a mathematical framework that has been used to think of the role of population structure in evolutionary dynamics. More recently, empirical scientists have become interested in this framework, but in most of the systems in their focus, the nodes are subpopulations and not individuals. Here, we have classified different classes of update mechanisms on such graph-structured metapopulations. We focus on update mechanisms that are natural extensions of the update mechanisms typically used in evolutionary graph theory for graphs of individuals.

Our classification is based on three factors:

(i) first, if migration is coupled to reproduction or not;
(ii) second, the order of birth and death events; and
(iii) third, how selection acts on the growth and survival of the population.

Each of these update mechanisms can result in different dynamics—using different update mechanisms can affect not only the fixation probability and fixation time of newly arising mutations but also other features of the dynamics.

The fixation probability in graphs of individuals under **Bd** (where selection for birth is proportional to a selection parameter) and **Db** (where selection for death is proportional to the inverse of the selection parameter for birth) are equivalent in undirected regular graphs [42] and they both follow the isothermal theorem [1]. For more details, see appendix A.2. In addition, in a fully connected graph of individuals, where all individuals are equivalent, and every node in the graph includes a self-loop, meaning that the individual selected for birth can die or the individual selected for death can give birth, **Bd** is equivalent to **dB**, **bD** is equivalent to **Db** and **BD** is equivalent to **DB**. It is worth mentioning that in an update mechanism where death is followed by birth, having self-loops in the graphs makes only limited sense if we think of the actual physical death of individuals. However, it is sensible if we think of it in the social setting where death and birth are interpreted as imitating one’s idea or sticking to your own [15]. Furthermore, in a fully connected graph including self-loops, if the selection parameter for death equals the inverse of the selection parameter for birth, then the fixation probabilities in the update mechanisms **Bd**, **dB**, **bD** and **Db** and are the same as the fixation probability of the well-mixed population under the update mechanism **Bd** [44]. However, in this condition, the fixation probability of a beneficial mutant in an arbitrary graph under **BD** and **DB** is higher than the respective fixation probability in the corresponding well-mixed population under **Bd**.

In a system where individuals with a higher selection parameter for birth have a lower selection parameter for death, the more the birth and death are associated with these selection parameters, the higher the probability for advantageous individuals to take over the population. This implies that the fixation probability of a beneficial mutant under **BD** is higher than the corresponding fixation probability under **Bd** and **bD**. Also the fixation probability of a beneficial mutant under **DB** is higher than the corresponding fixation probability under **Db** and **dB**. In addition, the fixation probability of a beneficial mutant in an arbitrary graph under an update mechanism in which selection is global is more than or equal to its fixation probability under an update mechanism in which selection is local [17]. Equality holds for a well-mixed population which includes self-loops meaning that every individual can also replace itself.

In a graph of subpopulations with update mechanisms where migration is coupled with death or birth, the fixation probability of an advantageous mutant in an arbitrary graph is higher under some update mechanisms compared with others. In appendix B, we consider two update mechanisms that are exactly the same except that the individual selection for birth or death in one is uniformly random and in the other is random proportional to selection parameters. The fixation probability of an advantageous mutant for selection proportional to selection parameters is higher than the fixation probability where selection is uniformly at random. For example, the update mechanism **MBBDD** has a higher fixation probability for advantageous mutants than **MBbDD**. In addition, one intuitively expects that if selection on the patch level is associated with a collective selection parameter, the beneficial mutant has a higher chance of being fixed. Nevertheless, it is not straightforward to prove this. In appendix B, we show this in more detail.

Similarly, as it is shown in appendix B, for the update mechanisms with uncoupled migration, if we have two update mechanisms that are only different in individual selection on birth or death, the fixation probability of an advantageous mutant under the update mechanism in which individual selection is uniformly random is smaller than the corresponding fixation probability under the update mechanism in which individual selection is associated with selection parameters. For instance, the fixation probability of an advantageous mutant under **mBBD** is higher than the corresponding fixation probability under **mBBd** in an arbitrary graph.

In addition, it is interesting to see under which of the coupled or uncoupled update mechanisms the beneficial mutant has a higher chance of taking over the whole population. Intuitively, under the coupled update mechanism, migration helps to spread beneficial mutants, whereas under uncoupled migration, exchanges of the individual between the patches occur uniformly at random and independent of the selection parameters.

In appendix D, we investigate the fixation probability for the star-structured metapopulation (metastar) under update mechanisms with coupled migration. The analysis is done in the low migration rate regime where every node (subpopulation) is in a homogeneous state at the time of migration. In the low migration rate regime, selection on the individual level plays an important role, whereas selection on the patch level changes the fixation probability only slightly. This leads to grouping of the update mechanisms into three classes based on the fixation probability of an advantageous mutant: update mechanisms in which both birth and death on the individual level are associated with the selection parameters, update mechanisms in which either birth or death on the individual level is associated with the selection parameter, and update mechanisms in which neither birth and nor death on the individual level are associated with the selection parameters.

Various update mechanisms have been applied to study biological, ecological and social systems. It has been argued that death–birth processes can be applied to study the evolutionary dynamics of trees in a tropical forest: a new seed grows into an adult tree when one of the trees in the forest dies [66]. Here, death happens first. Similarly, birth–death processes have been employed in cancer evolution [67,68]. Cancer cells grow excessively, and since they exhaust the nutrients and resources, the healthy cells die due to the lack of resources and leave empty spaces for the cancer cells to grow further. The evolutionary dynamics of the cancer cells arising in the crypts of the inner lining of the intestine can be modelled by a birth–death process in a line structure [69,70]. In this case, birth triggers death.

In a graph of subpopulations, coupled migration could denote the natural tendency of the species to look for a better place to live. Uncoupled migration can describe dispersal caused by humans or abiotic factors such as wind or water streams. The selection pressure on the patch level makes sense when patches share common and limited resources but still are partly isolated.

We hope that this paper paves the way for future work on the evolutionary dynamics of graph-structured metapopulations. Here, we only classify possible update mechanisms on metapopulations and only partly analyse some of them. However, the update mechanism is a crucial ingredient of evolutionary graph theory, and a better understanding of how it affects evolutionary dynamics in structured metapopulations will be necessary to move the field forward.

## Figures and Tables

**Figure 5.**
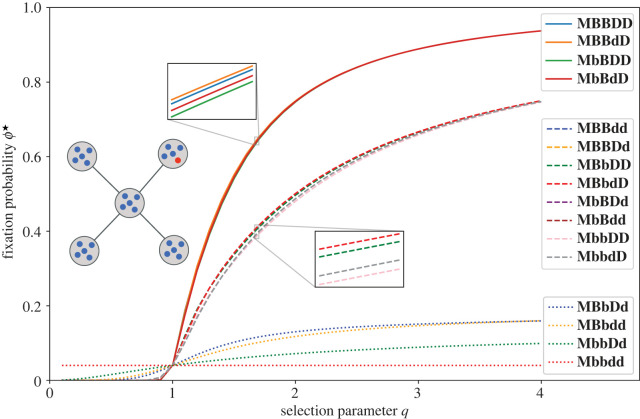
The fixation probability of the metastar in the low migration rate regime under the coupled update mechanisms with birth being first, starting from a single mutant. In terms of the value of fixation probability, one can put the update mechanisms into three classes: (H) a class of four update mechanisms with high fixation probability in which selection on the individual level both in both birth and death is associated with the selection parameter (solid lines), (I) a class of eight update mechanisms with medium fixation probability in which selection on the individual level is associated with the selection parameter either for birth or death (dashed lines), and (L) a class of four update mechanisms with low fixation probability in which selection on the individual level is uniformly random for both birth and death (dotted lines). Parameters: number of patches, *M* = 5, patch size, *N*_1_ = 5.

**Figure 6.**
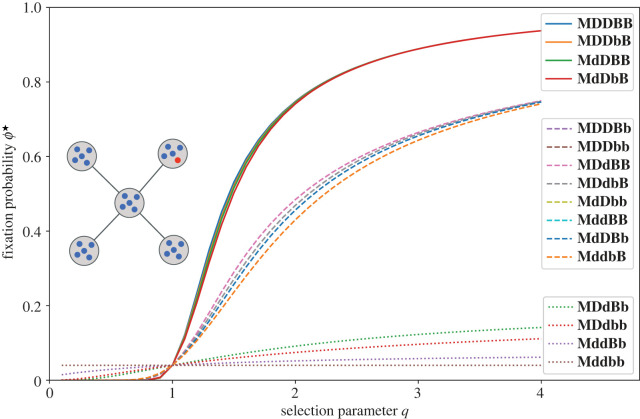
The fixation probability of the metastar in the low migration rate regime under coupled update mechanisms with death being first, starting from a single mutant. In terms of the value of fixation probability, one can again put the update mechanisms into three classes: (H) the class with high fixation probability in which selection on the individual level both in both birth and death is associated with the selection parameter, (I) the class with medium fixation probability in which selection on the individual level is associated with the selection parameter either for birth or death, and (L) the class with the low fixation probability in which selection on the individual level is uniformly random for both birth and death. Parameters: number of patches, *M* = 5, patch size, *N*_1_ = 5.

## Data Availability

This article has no additional data.

## Appendix A. Evolutionary dynamics on graphs of individuals

Assume a connected graph of individuals where *w*_*ij*_ is the weight of the link connecting node *i* to *j*. The population consists of two types, wild-type A and mutant B. The variable *s*_*i*_ indicates the status of node *i*, *s*_*i*_ = 0 if it is occupied by a wild-type, and *s*_*i*_ = 1 if it is occupied by a mutant. The selection parameter for the birth of the mutant with respect to the wild-type is *r*, and the selection parameter for the death of the mutant with respect to the wild-type is *t*. In the following section, we explain the transition probabilities for both birth–death and death–birth processes on graphs of individuals.

**A.1. Transition probabilities**

In an update mechanism where birth is global and death is local, one individual is selected for birth, and then one of its neighbours is chosen for death. The offspring will replace the empty spot. Based on this model, there are two possible transitions: increasing and decreasing the number of mutants by one. The probability $T_{\mathrm{birth}-\mathrm{death}}^{n+}$ of increasing the number of mutants, $n=\sum_{i}s_{i}$, by one is

A.1 $$T_{\mathrm{birth}-\mathrm{death}}^{n+}=\sum_{i}\underset{\mathrm{birth}}{\underbrace{\frac{rs_{i}}{r\sum_{k}s_{k}+\sum_{k}(1-s_{k})}}}\underset{\mathrm{death}}{\underbrace{\frac{\sum_{j}w_{ij}(1-s_{j})}{t\sum_{l}w_{il}s_{l}+\sum_{l}w_{il}(1-s_{l})}}}.$$

This equation is the summation over all the possibilities that the number of mutants increases. The probability $T_{\mathrm{birth}-\mathrm{death}}^{n-}$ of decreasing the number of mutants *n* by one is

A.2 $$T_{\mathrm{birth}-\mathrm{death}}^{n-}=\sum_{i}\underset{\mathrm{birth}}{\underbrace{\frac{1-s_{i}}{r\sum_{k}s_{k}+\sum_{k}(1-s_{k})}}}\underset{\mathrm{death}}{\underbrace{\frac{t\sum_{j}w_{ij}s_{j}}{t\sum_{l}w_{il}s_{l}+\sum_{l}w_{il}(1-s_{l})}}}.$$

When death is global and birth is local, an individual is selected for death, and then from its neighbour, one individual is selected for birth. In this case the transition probabilities are

A.3 $$T_{\mathrm{death}-\mathrm{birth}}^{n+}=\sum_{i}\underset{\mathrm{death}}{\underbrace{\frac{1-s_{i}}{t\sum_{k}s_{k}+\sum_{k}(1-s_{k})}}}\underset{\mathrm{birth}}{\underbrace{\frac{r\sum_{\;j}w_{ij}s_{j}}{r\sum_{l}w_{il}s_{l}+\sum_{l}w_{il}(1-s_{l})}}}$$

and

A.4 $$T_{\mathrm{death}-\mathrm{birth}}^{n-}=\sum_{i}\underset{\mathrm{death}}{\underbrace{\frac{ts_{i}}{t\sum_{k}s_{k}+\sum_{k}(1-s_{k})}}}\underset{\mathrm{birth}}{\underbrace{\frac{\sum_{\;j}w_{ij}(1-s_{j})}{r\sum_{l}w_{il}s_{l}+\sum_{l}w_{il}(1-s_{l})}}}.$$

Based on the values of *r* and *t*, the update mechanisms can be categorized as follows:

(i) In the above equations if *r* ≠ 1 and *t* ≠ 1 there are selection pressures both on the birth and the death. This corresponds to the update mechanism **BD** in which birth is global and death is local, and the update mechanism **DB** in which death is global and death is local.
(ii) If *r* ≠ 1 and *t* = 1 birth–death corresponds to update mechanism **Bd** and death–birth corresponds to **dB**.
(iii) If *r* = 1 and *t* ≠ 1, birth–death correspond to **bD** and death–birth correspond to **Db**.
(iv) If *r* = 1 and *t* = 1 birth–death corresponds to **bd** and death–birth corresponds to **db**.

**A.2. Equivalence of **Bd** and **Db** in undirected regular graphs**

Generalizing the transition probabilities from appendix A.1, to an arbitrary weighted graph with weights *w*_*ij*_ under the update mechanisms **Bd** leads to

A.5 $$T_{\mathrm{Bd}}^{n+}=\sum_{i}\underset{\mathrm{birth}\;}{\underbrace{\frac{rs_{i}}{r\sum_{k}s_{k}+\sum_{k}(1-s_{k})}}}\underset{\mathrm{death}}{\underbrace{\frac{\sum_{j}w_{ij}(1-s_{j})}{\sum_{l}w_{il}}}}$$

and

A.6 $$T_{\mathrm{Bd}}^{n-}=\sum_{i}\underset{\mathrm{birth}}{\underbrace{\frac{1-s_{i}}{r\sum_{k}s_{k}+\sum_{k}(1-s_{k})}}}\underset{\mathrm{death}}{\underbrace{\frac{\sum_{j}w_{ij}s_{j}}{\sum_{l}w_{il}}}}.$$

The transition probabilities of an arbitrary graph under the update mechanisms **Db** are

A.7 $$T_{\mathrm{Db}}^{n+}=\sum_{i}\underset{\mathrm{death}}{\underbrace{\frac{1-s_{i}}{t\sum_{k}s_{k}+\sum_{k}(1-s_{k})}}}\underset{\mathrm{birth}}{\underbrace{\frac{\sum_{j}w_{ij}s_{j}}{\sum_{l}w_{il}}}}$$

and

A.8 $$T_{\mathrm{Db}}^{n-}=\sum_{i}\underset{\mathrm{death}}{\underbrace{\frac{ts_{i}}{t\sum_{k}s_{k}+\sum_{k}(1-s_{k})}}}\underset{\mathrm{birth}}{\underbrace{\frac{\sum_{j}w_{ij}(1-s_{j})}{\sum_{l}w_{il}}}}.$$

In a regular graph, $\sum_{l}w_{il}$ is identical for all the nodes. Thus we can set it as $\sum_{l}w_{il}=\alpha$. As a result, the transition probabilities for update mechanism **Bd** are simplified to

A.9 $$T_{\mathrm{Bd}}^{n+}=\frac{r}{\alpha (rn+N-n)}\sum_{i,j}w_{ij}s_{i}(1-s_{j})\;$$

and

A.10 $$T_{\mathrm{Bd}}^{n-}=\frac{1}{\alpha (rn+N-n)}\sum_{i,j}w_{ij}(1-s_{i})s_{j}.$$

Since in the regular graphs *w*_*ij*_ = *w*_*ji*_, therefore, $\sum_{i,j}w_{ij}(1-s_{i})s_{j}=$ $\sum_{i,j}w_{ij}(1-s_{\;j})s_{i}$ and consequently $T_{\mathrm{Bd}}^{n-}/T_{\mathrm{Bd}}^{n+}=1/r$ is independent of the *s*_*i*_. As a result the fixation probability is $\phi^{\left(n\right)}=\frac{1-1/r^{n}}{1-1/r^{N}}$.

Similarly, the transition probabilities for the update mechanism **Db** simplify to

A.11 $$T_{\mathrm{Db}}^{n+}=\frac{1}{\alpha (tn+N-n)}\sum_{i,j}w_{ij}(1-s_{i})s_{j}$$

and

A.12 $$T_{\mathrm{Db}}^{n-}=\frac{t}{\alpha (tn+N-n)}\sum_{i,j}w_{ij}s_{i}(1-s_{j}).$$

Since $T_{\mathrm{Db}}^{n-}/T_{\mathrm{Db}}^{n+}=t$, the fixation probability is $\phi^{\left(n\right)}=\frac{1-t^{n}}{1-t^{N}}$; cf. equation (2.3). If we set *t* = 1/*r* the fixation probability is the same as the fixation probability of the equivalent well-mixed population under the update mechanism **Bd**.

## Appendix B. Evolutionary dynamics on graphs of subpopulations

This appendix discusses why some update mechanisms fix beneficial mutants with higher probability. Assume that we have two types of individuals, mutants and wild-types. The transition probabilities of increasing and decreasing the total number of mutants *n* are given by *T*^*n*+^ and *T*^*n*−^, respectively. We use a mean-field approximation and assume that the transition probabilities are the summation of the transition probabilities for all the possible configurations for a specific number of mutants *n*. The other parameters are described in table 4.

**Table 4.** Parameters for a graph of subpopulations.

parameter	description
*N*_*i*_	population size in patch *i*
*n*_*i*_	number of mutants in patch *i*
*n*	total number of mutants, $\sum_{i}n_{i}$
*N*	total population size, $\sum_{i}N_{i}$
*r*	selection parameter for birth of the mutant
*t*	selection parameter for death of the mutant
*λ*	migration probability
*w*_*ij*_	weight of the link from patch *i* to patch *j*

If we start with a single randomly placed mutant in a wild-type population, the fixation probability of the mutant is given by [24]

B.1 $$\phi^{\left(1\right)}=\frac{1}{1+\sum_{i=1}^{N-1}\prod_{\;j=1}^{i}(T^{\;j-}/T^{\;j+})}.$$

Therefore, in order to investigate how the fixation probabilities in different update rules vary, it is sufficient to compare the transition probabilities.

**B.1. Comparison of update mechanism with or without individual-level selection**

By comparing the transition probabilities, we can see that for both coupled and uncoupled update mechanisms, if two update mechanisms only differ in individual-level selection for either birth or death, the fixation probability of the beneficial mutant under the update mechanism in which individual selection is associated with selection parameter is higher than the one in which individual selection is uniformly random. Here, we compare the transition probabilities of update mechanisms **MBBDD**, **MBbDD**, **MBBDd**. The transition probabilities of increasing and decreasing the total number of mutants, $n=\sum_{i}n_{i}$ update mechanism **MBBDD** are

B.2 TMBBDDn+=∑irni+Ni−nirn+N−n⏟birth patchrnirni+Ni−ni⏟birth of individual×(λ∑jwijtnj+Nj−nj∑kwik(tnk+Nk−nk)⏟choosing a patch to migrate toNj−njtnj+Nj−nj⏟death of individual+(1−λ)Ni−nitni+Ni−ni⏟death of individual in parental patch)

and

B.3 $$\;\begin{array}{rl} & T_{\mathrm{MBBDD}}^{n-}=\sum_{i}\underset{\mathrm{selection}\;\mathrm{patch}\;\mathrm{birth}}{\underbrace{\frac{rn_{i}+N_{i}-n_{i}}{rn+N-n}}}\underset{\mathrm{birth}\;\mathrm{of}\;\mathrm{individual}}{\underbrace{\frac{N_{i}-n_{i}}{rn_{i}+N_{i}-n_{i}}}}\\ & \times (\lambda \underset{\mathrm{choosing}\;a\;\mathrm{patch}\;\mathrm{to}\;\mathrm{migrate}\;\mathrm{to}}{\underbrace{\sum_{j}w_{ij}\frac{tn_{j}+N_{j}-n_{j}}{\sum_{k}w_{ik}(tn_{k}+N_{k}-n_{k})}}}\underset{\mathrm{death}\;\mathrm{of}\;\mathrm{individual}}{\underbrace{\frac{tn_{j}}{tn_{j}+N_{j}-n_{j}}}}\\ & \;+(1-\lambda )\underset{\begin{array}{c} \mathrm{death}\;\mathrm{of}\;\mathrm{individual}\\ \;\mathrm{in}\;\mathrm{parental}\;\mathrm{patch} \end{array}}{\underbrace{\frac{tn_{i}}{tn_{i}+N_{i}-n_{i}}}}).\; \end{array}$$

The transition probabilities for the update mechanism **MBbDD** are

B.4 $$\;\begin{array}{rl} T_{\mathrm{MBbDD}}^{n+} & =\sum_{i}\frac{rn_{i}+N_{i}-n_{i}}{rn+N-n}\frac{n_{i}}{N_{i}}\\ & \;\times (\lambda \sum_{j}w_{ij}\frac{tn_{j}+N_{j}-n_{j}}{\sum_{k}w_{ik}(tn_{k}+N_{k}-n_{k})}\frac{N_{j}-n_{j}}{tn_{j}+N_{j}-n_{j}}\\ & \;+(1-\lambda )\frac{N_{i}-n_{i}}{tn_{i}+N_{i}-n_{i}})\; \end{array}$$

and

B.5 $$\;\begin{array}{rl} T_{\mathrm{MBbDD}}^{n-} & =\sum_{i}\frac{rn_{i}+N_{i}-n_{i}}{rn+N-n}\frac{N_{i}-n_{i}}{N_{i}}\\ & \;\times (\lambda \sum_{j}w_{ij}\frac{tn_{j}+N_{j}-n_{j}}{\sum_{k}w_{ik}(tn_{k}+N_{k}-n_{k})}\frac{tn_{j}}{tn_{j}+N_{j}-n_{j}}\\ & \;+(1-\lambda )\frac{tn_{i}}{tn_{i}+N_{i}-n_{i}}).\; \end{array}$$

The transition probabilities for the update mechanism **MBBDd** are

B.6 $$\begin{array}{rl} & T_{\mathrm{MBBDd}}^{n+}=\sum_{i}\frac{rn_{i}+N_{i}-n_{i}}{rn+N-n}\frac{rn_{i}}{rn_{i}+N_{i}-n_{i}}\\ & \;\times (\lambda \sum_{j}w_{ij}\frac{tn_{j}+N_{j}-n_{j}}{\sum_{k}w_{ik}(tn_{k}+N_{k}-n_{k})}\frac{N_{j}-n_{j}}{N_{j}}+(1-\lambda )\frac{N_{i}-n_{i}}{N_{i}}) \end{array}$$

and

B.7 $$\begin{array}{rl} T_{\mathrm{MBBDd}}^{n-} & =\sum_{i}\frac{rn_{i}+N_{i}-n_{i}}{rn+N-n}\frac{N_{i}-n_{i}}{rn_{i}+N_{i}-n_{i}}\\ & \;\times (\lambda \sum_{j}w_{ij}\frac{tn_{j}+N_{j}-n_{j}}{\sum_{k}w_{ik}(tn_{k}+N_{k}-n_{k})}\frac{n_{j}}{N_{j}}+(1-\lambda )\frac{n_{i}}{N_{i}}). \end{array}$$

Comparing equations (B.2) and (B.4), all the terms are the same except the second term which is the probability of choosing a mutant in patch *i* for birth. Since

B.8 $$\frac{rn_{i}}{rn_{i}+N_{i}-n_{i}}>\frac{n_{i}}{N_{i}},$$

for beneficial mutants, *r* > 1 (except for *n*_*i*_ = *N*_*i*_ and *n*_*i*_ = 0, where the transition probabilities are zero) that implies

B.9 $$T_{\mathrm{MBBDD}}^{n+}>T_{\mathrm{MBbDD}}^{n+}.$$

Also if we compare equations (B.3) and (B.5) since

B.10 $$\frac{N_{i}-n_{i}}{rn_{i}+N_{i}-n_{i}}<\frac{N_{i}-n_{i}}{N_{i}},$$

for beneficial mutants, *r* > 1 (except for *n*_*i*_ = *N*_*i*_ and *n*_*i*_ = 0, where the transition probabilities are zero). Thus, we find

B.11 $$T_{\mathrm{MBBDD}}^{n-}<T_{\mathrm{MBbDD}}^{n-}.$$

As a result,

B.12 $$\frac{T_{\mathrm{MBBDD}}^{n-}}{T_{\mathrm{MBBDD}}^{n+}}<\frac{T_{\mathrm{MBbDD}}^{n-}}{T_{\mathrm{MBbDD}}^{n+}}\;\text{ for all}\;1<n<N-1.$$

The ratio *T*^*n*−^/*T*^*n*+^ appears in the denominator of equation (B.1). This implies that the fixation probability of an advantageous mutant under **MBBDD** is higher than the corresponding fixation probability under **MBbDD** for an arbitrary graph, *ϕ*_MBBDD_ > *ϕ*_MBbDD_. Similarly, we can show that the fixation probability of a deleterious mutant under **MBBDD** is lower than the corresponding fixation probability under **MBbDD** for an arbitrary graph, *ϕ*_MBBDD_ < *ϕ*_MBbDD_.

In addition, comparing equations (B.2) and (B.6), since

B.13 $$\frac{N_{j}-n_{j}}{tn_{j}+N_{j}-n_{j}}>\frac{N_{j}-n_{j}}{N_{j}},$$

for beneficial mutants, *t* < 1, we have

B.14 $$T_{\mathrm{MBBDD}}^{n+}>T_{\mathrm{MBBDd}}^{n+}.$$

Also by comparing equations (B.3) and (B.7), we have

B.15 $$T_{\mathrm{MBBDD}}^{n-}<T_{\mathrm{MBBDd}}^{n-}$$

because

B.16 $$\frac{dn_{j}}{dn_{j}+N_{j}-n_{j}}<\frac{n_{j}}{N_{j}},$$

for all 1 < *n*_*j*_ < *N*_*j*_ − 1 and for *n*_*j*_ = *N*_*j*_ both sides are equal. In conclusion, the fixation probability of an advantageous mutant in an arbitrary graph under **MBBDd** is smaller than the corresponding fixation probability under **MBBDD**. On the other hand, for deleterious mutants, $\phi_{\mathrm{MBBDd}}>\phi_{\mathrm{MBBDD}}$.

We can show in a similar way as above that for beneficial mutants that the fixation probability, *ϕ* of an arbitrary graph under update mechanism **MBBDD**, **MBbDD** and **MBbDd** have the following relationship with each other:

B.17 $$\phi_{\mathrm{MBBDD}}>\phi_{\mathrm{MBbDD}}>\phi_{\mathrm{MBbDd}},$$

and similarly the relation between the fixation probability of a beneficial mutant for an arbitrary graph under update mechanisms **MBBDD**, **MBBDd** and **MBbDd** is

B.18 $$\phi_{\mathrm{MBBDD}}>\phi_{\mathrm{MBBDd}}>\phi_{\mathrm{MBbDd}}.$$

In the above expressions, one cannot simply state which of the *ϕ*_MBBDd_ and *ϕ*_MBbDD_ is higher. The relation between these two values might be dependent on the graph structure.

On the other hand, the relation between the fixation probabilities, *ϕ*, of a deleterious mutant in an arbitrary graph under the update mechanisms, **MBBDD**, **MBbDD** and **MBbDd** is

B.19 $$\phi_{\mathrm{MBBDD}}<\phi_{\mathrm{MBbDD}}<\phi_{\mathrm{MBbDd}},$$

and similarly, we can simply show that the fixation probabilities of a deleterious mutant in an arbitrary graph under the update mechanisms **MBBDD**, **MBBDd** and **MBbDd** has the following relationship:

B.20 $$\phi_{\mathrm{MBBDD}}<\phi_{\mathrm{MBBDd}}<\phi_{\mathrm{MBbDd}}.$$

As we see from these equations, for an arbitrary graph, if an update mechanism is more associated with selection parameters, the fixation probability of advantageous mutants increases and the fixation probability of deleterious mutants decreases.

**B.2. Comparison of update mechanism with or without patch level selection**

Comparing the transition probabilities of a beneficial mutant under two update mechanisms that only differ in patch level selection for either birth or death is not as straightforward. Intuitively we expect that the update mechanism in which patch level selection is associated with the collective selection parameter of the patch has a higher fixation probability. In the following, we show why one cannot easily infer from the transition probabilities which one is higher. Let us compare the transition probabilities for increasing the mutant population size by one under the update mechanisms **MBBDD** and **MbBDD**.

B.21 $$\;\begin{array}{rl} T_{\mathrm{MbBDD}}^{n+} & =\sum_{i}\underset{\mathrm{birth}\;\mathrm{patch}}{\underbrace{\frac{N_{i}}{N}}}\underset{\mathrm{birth}\;\mathrm{of}\;\mathrm{individual}}{\underbrace{\frac{rn_{i}}{rn_{i}+N_{i}-n_{i}}}}\\ & \;\times (\lambda \underset{\mathrm{choosing}\;a\;\mathrm{patch}\;\mathrm{to}\;\mathrm{migrate}\;\mathrm{to}}{\underbrace{\sum_{j}w_{ij}\frac{tn_{j}+N_{j}-n_{j}}{\sum_{k}w_{ik}(tn_{k}+N_{k}-n_{k})}}}\underset{\mathrm{death}\;\mathrm{of}\;\mathrm{individual}}{\underbrace{\frac{N_{j}-n_{j}}{tn_{j}+N_{j}-n_{j}}}}\\ & \;+(1-\lambda )\underset{\begin{array}{c} \mathrm{death}\;\mathrm{of}\;\mathrm{individual}\\ \;\mathrm{in}\;\mathrm{parental}\;\mathrm{patch} \end{array}}{\underbrace{\frac{N_{i}-n_{i}}{tn_{i}+N_{i}-n_{i}}}})\; \end{array}$$

Comparing the equations (B.2) and (B.21), the only difference is the term for the birth patch. $T_{\mathrm{MBBDD}}^{n+}>T_{\mathrm{MbBDD}}^{n+}$ if for all *i* values

B.22 $$\frac{rn_{i}+N_{i}-n_{i}}{rn+N-n}\geq \frac{N_{i}}{N}.$$

The above relation holds if and only if

B.23 $$\frac{n_{i}}{N_{i}}\geq \frac{n}{N}.$$

However, the above relation does not always hold, and it depends on the configuration of the population. Hence, it is not easy to compare equations (B.2) and (B.21).

**B.3. Comparison of update mechanism with uncoupled migration**

Similarly, by comparing the transition probabilities for uncoupled update mechanisms, we can see that the fixation probability of an advantageous mutant under update mechanisms in which individual selection is associated with selection parameters is higher than the corresponding fixation probability under update mechanisms in which individual selection is independent of selection parameters. The transition probabilities of this update mechanism only include the non-migrative terms because the migrative term does not change the number of mutants in the whole population. The transition probabilities for the update mechanism **mBBD** are

B.24 $$T_{\mathrm{mBBD}}^{n+}=(1-\lambda )\sum_{i}\frac{rn_{i}+N_{i}-n_{i}}{rn+N-n}\frac{rn_{i}}{rn_{i}+N_{i}-n_{i}}\frac{N_{i}-n_{i}}{tn_{i}+N_{i}-n_{i}}\;$$

and

B.25 $$T_{\mathrm{mBBD}}^{n-}=(1-\lambda )\sum_{i}\frac{rn_{i}+N_{i}-n_{i}}{rn+N-n}\frac{N_{i}-n_{i}}{rn_{i}+N_{i}-n_{i}}\frac{tn_{i}}{tn_{i}+N_{i}-n_{i}},$$

and the transition probabilities for the update mechanism **mBBd** are

B.26 $$T_{\mathrm{mBBd}}^{n+}=(1-\lambda )\sum_{i}\frac{rn_{i}+N_{i}-n_{i}}{rn+N-n}\frac{rn_{i}}{rn_{i}+N_{i}-n_{i}}\frac{N_{i}-n_{i}}{N_{i}}\;$$

and

B.27 $$T_{\mathrm{mBBd}}^{n-}=(1-\lambda )\sum_{i}\frac{rn_{i}+N_{i}-n_{i}}{rn+N-n}\frac{N_{i}-n_{i}}{rn_{i}+N_{i}-n_{i}}\frac{n_{i}}{N_{i}}.$$

From equations (B .24) and (B. 26), we can see that for beneficial mutants, *t* < 1,

B.28 $$T_{\mathrm{mBBD}}^{n+}>T_{\mathrm{mBBd}}^{n+},$$

because for 1 < *n*_*i*_ < *N*_*i*_ we have

B.29 $$\frac{N_{i}-n_{i}}{dn_{i}+N_{i}-n_{i}}>\frac{N_{i}-n_{i}}{N_{i}}.$$

Analogously, for the beneficial mutants we have

B.30 $$T_{\mathrm{mBBD}}^{n-}<T_{\mathrm{mBBd}}^{n-}.$$

Thus, the fixation probability of an advantageous mutant under **mBBD** is higher than the corresponding fixation probability under **mBBd**, $\phi_{\mathrm{mBBD}}>\phi_{\mathrm{mBBd}}$. Similarly we can see that for a beneficial mutant

B.31 $$\phi_{\mathrm{mBbd}}<\phi_{\mathrm{mBbd}}<\phi_{\mathrm{mBBD}}.$$

On the other hand, for the deleterious mutants we have an opposite relation between the fixation probabilities:

B.32 $$\phi_{\mathrm{mBbd}}>\phi_{\mathrm{mBbd}}>\phi_{\mathrm{mBBD}}.$$

## Appendix C. Equivalence of evolutionary dynamics on graphs of subpopulations and graphs of individuals

In the mechanisms with coupled migration, some update mechanisms reduce to simpler ones in a weighted graph of individuals, where the weights of the links that connect individuals locally are different from the weights of the links that connect individuals in adjacent subpopulations (see figure 4).

In order for an update mechanism to reduce to a simpler update mechanism, selection for birth and death should be either associated with selection parameter or not for both patch and individual levels. As an example **MBBdd** reduces to **Bd**. This can be easily shown by transition probabilities; in order to increase the number of mutants by one through selecting one mutant from the patch *i* consists of two parts; first selecting a mutant from patch *i* with probability

C.1 $$\frac{rn_{i}+N_{i}-n_{i}}{rn+N-n}\frac{r}{rn_{i}+N_{i}-n_{i}}.$$

This probability is simplified to *r*/(*rn* + *N* − *n*), which is equivalent to the probability of selecting one mutant from the whole population regardless of the collective selection parameter of the patches. The second part is choosing one of the wild-type neighbours of the selected mutant for death. The neighbour could be either selected from the parental patch with probability

C.2 $$(1-\lambda )\frac{N_{i}-n_{i}}{N_{i}},$$

or from the neighbouring patches with probability

C.3 $$\lambda \sum_{j}w_{ij}\frac{N_{j}-n_{j}}{\sum_{k}w_{ik}N_{k}}.$$

The above two equations for the death probability of a wild-type imply that a graph of patches can be reduced to a graph of individuals. In the equivalent graph of individuals the weight of the link between each two individuals within the patch *i* is (1 − *λ*)/*N*_*i*_ if we take into account self-loops, and the weight of the link from an individual from patch *i* to an individual from patch *j* is $\lambda w_{ij}/\sum_{j}w_{ij}N_{j}$. Therefore, the update mechanism **MBBdd** on a graph of subpopulations is equivalent to the update mechanism **Bd** on a graph of individuals in which the weight of the links that connect local individuals differ from the weight of links that connect individuals in different patches. The weight of the links depends on the migration probability as well as the local population sizes.

## Appendix D. Evolutionary dynamics on the metastar

In this appendix, we calculate the fixation probability for the metastar in the low migration rate regime. A metastar is a star graph where every node is occupied by a subpopulation. The internal structure of each subpopulation is well mixed. These subpopulations are connected to each other through the central node. We denote the total population size by *N* and assume it is distributed evenly into *M* patches, so that the size of each patch is *N*_1_ = *N*/*M*.

When the migration probability is sufficiently low, and the population sizes of the patches are comparable, the individual migrating to a neighbouring patch either reaches fixation or goes extinct in that respective patch before the next migration event occurs. Therefore, the subpopulations are in a homogeneous configuration at the time of a migration event. By homogeneous configuration, we mean that each patch is either occupied by the mutant type or the wild-type individuals. In the low migration rate regime, to calculate the fixation probability, instead of considering the transition probabilities between 2^*N*^ states, the state space is reduced to 2^*M*^. The transition probabilities between these 2^*M*^ states are sufficient to compute fixation probabilities. As a result, we can look at the problem as if we have a graph of individuals. However, the probability of replacing a node with the offspring of a neighbouring node needs to be modified. In a graph of individuals, the probability that the offspring of a chosen node *i* replaces the individual of node *k* is equal to the weight of the link directed from node *i* to node *k*, *w*_*ik*_. In a graph of subpopulations, assuming the low migration rate regime, this probability is modified to $w_{ik}\phi_{\mathrm{wm}}^{N_{k}}$, where $\phi_{\mathrm{wm}}^{N_{k}}$ is the fixation probability of the migrating offspring to take over the patch *k* of size *N*_*k*_, and wm stands for well-mixed. With the individual-selection parameters *r* and *t*, we obtain from equation (B.1)

D.1 $$\phi_{\mathrm{wm}}^{N_{k}}=\frac{1-(t/r)}{1-{\left(t/r\right)}^{N_{k}}}.$$

Here, we focus on the update mechanisms with coupled migration described in §3.1.

**D.1. Update mechanisms with birth first**

We start with writing the transition probabilities for all the 16 update mechanisms in table 2 in a compact way. To do so, in addition to the selection parameters *r* and *t* introduced in table 4, we introduce two more parameters *r*′ and *t*′. Parameters *r* and *t* represent selection at the individual level for birth and death, respectively, whereas, parameters *r*′ and *t*′ represent selection at the patch level for birth and death, respectively. If the selection for birth at the individual level is uniformly at random, then *r* = 1, otherwise *r* ≠ 1. Similarly, if the selection for death at the individual level is uniformly at random, then *t* = 1, otherwise *t* ≠ 1. The same holds for the parameters defined at the patch level. As an example, for the update mechanism **MbBDD**, since the birth selection at the patch level is uniformly random, *r*′ = 1. But the parameters *r*, *t* and *t*′ are not equal to 1. The parameters *r*′ and *t*′ describe the contribution of an individual to the collective selection parameters of a patch. When a patch is occupied by mutants, its collective selection parameters for the birth and the death events equal *r*′*N*_1_ and *t*′*N*_1_, respectively. When a patch is occupied by wild-types, its collective selection parameters for the birth and the death events equal *N*_1_. With this in mind, we generalize the transition probabilities for update mechanisms with coupled migration, with birth being the first event.

A state of the metastar is denoted by (∙/∘,j), where the first index represents the state of the central patch: ∙ if the central node is occupied by mutant individuals, ∘ if it is occupied by wild-type individuals. The second index denotes the number of mutant occupied leaf patches. The transition from the state (∙,j) to the state (∙,j+1) occurs with probability

D.2 $$T_{∙\to ∙}^{\;j+}=\frac{r^{\prime }N_{1}}{(j+1)r^{\prime }N_{1}+(M-1-j)N_{1}}\frac{(M-1-j)N_{1}}{(M-1-j)N_{1}+jt^{\prime }N_{1}}\lambda \phi_{\mathrm{wm}}^{N_{1}}(r,t).$$

The transition from the state (∘,j) to the state (∙,j) occurs with probability

D.3 $$T_{\circ \to ∙}^{\;j}=\frac{\;jr^{\prime }N_{1}}{jr^{\prime }N_{1}+(M-j)N_{1}}\lambda \phi_{\mathrm{wm}}^{N_{1}}(r,t).$$

The transition from the state (∘,j) to the state (∘,j−1) occurs with probability

D.4 $$T_{\circ \to \circ }^{\;j-}=\frac{N_{1}}{jr^{\prime }N_{1}+(M-j)N_{1}}\frac{\;jt^{\prime }N_{1}}{(M-1-j)N_{1}+jt^{\prime }N_{1}}\lambda \phi_{\mathrm{wm}}^{N_{1}}\left(\frac{1}{r},\frac{1}{t}\right).$$

The transition from the state (∙,j) to the state (∘,j) occurs with probability

D.5 $$T_{∙\to \circ }^{\;j}=\frac{(M-1-j)N_{1}}{(j+1)r^{\prime }N_{1}+(M-1-j)N_{1}}\lambda \phi_{\mathrm{wm}}^{N_{1}}\left(\frac{1}{r},\frac{1}{t}\right).$$

The fixation probability of a mutant taking over the entire metastar depends on the patch where the initial mutant appears. It can be a leaf patch or the central patch. Let us denote the fixation probability of a mutant starting from the central patch by $\phi_{∙}^{0}$. Using [6,25], we find

D.6 $$\phi_{∙}^{0}=\frac{\pi_{∙}^{0+}}{1+(1-\pi_{∙}^{0+})\sum_{j=1}^{M-2}{\left(\frac{\pi_{\circ }^{j-}}{\pi_{∙}^{j+}}\right)}^{j}}$$

Here, $\pi_{\circ }^{\;j-}$ is the conditional probability of the transition from the state (∘,   j) to the state (∘,   j−1) given that the population starts from state (○, *j*),

D.7 $$\pi_{\circ }^{j-}=\frac{T_{\circ \to \circ }^{j-}}{T_{\circ \to ∙}^{j}+T_{\circ \to \circ }^{j-}}=\frac{\frac{t^{\prime }}{r^{\prime }}\phi_{\mathrm{wm}}^{N_{1}}(1/r,1/t)}{\frac{t^{\prime }}{r^{\prime }}\phi_{\mathrm{wm}}^{N_{1}}(1/r,\;1/t)+(M-1-j+jt^{\prime })\phi_{\mathrm{wm}}^{N_{1}}(r,t)}.$$

Similarly, $\pi_{∙}^{\;j+}$ is the conditional probability of the transition from the state (∙,j) to the state (∙,j+1) given that the population starts from state (∙,j),

D.8 $$\pi_{∙}^{j+}=\frac{T_{∙\to ∙}^{j+}}{T_{∙\to ∙}^{j+}+T_{∙\to \circ }^{j}}=\frac{r^{\prime }\phi_{\mathrm{wm}}^{N_{1}}(r,t)}{r^{\prime }\phi_{\mathrm{wm}}^{N_{1}}(r,t)+(M-1-j+jt^{\prime })\phi_{\mathrm{wm}}^{N_{1}}(1/r,\;1/t)}.$$

The fixation probability of a mutant starting from a leaf patch, $\phi_{\circ }^{1}$, can be obtained by using the relation

D.9 $$\phi_{\circ }^{1}=\frac{1-\pi_{\circ }^{1-}}{\pi_{∙}^{1+}}\phi_{∙}^{0}.$$

Thus, the average fixation probability of a metastar starting from a single fully occupied mutant patch is,

D.10 $${\tilde{\phi }}^{⋆}=\frac{M-1}{M}\phi_{\circ }^{1}+\frac{1}{M}\phi_{∙}^{0}.$$

Consequently, the average fixation probability of a metastar staring from an individual mutant is

D.11 $$\phi^{⋆}=\;\;{\tilde{\phi }}^{⋆}\cdot \phi_{\mathrm{wm}}^{N_{1}}(r,t).$$

Using equations (D.6), (D.9) and (D.11), we find

D.12 $$\begin{array}{rl} \phi^{⋆} & =\left(\frac{M-1}{M}\phi_{\circ }^{1}+\frac{1}{M}\phi_{∙}^{0}\right)\phi_{\mathrm{wm}}^{N_{1}}(r,t),\\ & =\left(\frac{M-1}{M}\frac{1-\pi_{\circ }^{1-}}{\pi_{∙}^{1+}}+\frac{1}{M}\right)\frac{\pi_{∙}^{0+}}{1+(1-\pi_{∙}^{0+})\sum_{\;j=1}^{M-2}{\left(\frac{\pi_{\circ }^{j-}}{\pi_{∙}^{j+}}\right)}^{j}}\phi_{\mathrm{wm}}^{N_{1}}(r,t). \end{array}$$

To illustrate the fixation probability $\phi^{⋆}$ using equation (D.12), we assume that all selection parameters are controlled by the same variable, *q*. If any of the selection parameters for birth is not equal to 1, then it is set to *q*. That is, if *r* ≠ 1, then *r* = *q* and if *r*′ ≠ 1, then *r*′ = *q*. Similarly, if *t* ≠ 1, then *t* = 1/*q* and if *t*′ ≠ 1, then *t*′ = 1/*q*. Figure 5 shows the fixation probability of the metastar under different update schemes with migration coupled to the birth event. Based on the fixation probabilities for *q* > 1, the birth first update mechanisms with coupled migration can be broadly categorized into three classes with high, intermediate and low fixation probabilities:

(A) The class with high fixation probability consists of the update rules where selection acts at the individual level, both in the birth and the death event. That is in the high fixation probability class, *r* ≠ 1 and *t* ≠ 1.
(B) The class with intermediate fixation probability consists of the update mechanisms where selection acts at the individual level but only in one event, either birth (*r* ≠ 1) or death (*t* ≠ 1).
(C) The low fixation probability class consists of the update mechanisms where selection does not operate at the individual level. For this class, *r* = *t* = 1.

The ordering of the fixation probabilities corresponding to the update mechanisms of these three classes is consistent with the inequalities (B.17) and (B.18) obtained in appendix B.1. For example in inequality (B.17), it is shown that

D.13 $$\underset{\begin{array}{rl} & \;\mathrm{high}\;\mathrm{fixation}\\ & \mathrm{probability}\;\mathrm{class} \end{array}}{\underbrace{\phi_{MBBDD}}}>\underset{\begin{array}{rl} & \;\mathrm{intermediate}\;\mathrm{fixation}\\ & \;\mathrm{probability}\;\mathrm{class} \end{array}}{\underbrace{\phi_{MBBDd}}}>\underset{\begin{array}{rl} & \;\mathrm{low}\;\mathrm{fixation}\\ & \mathrm{probability}\;\mathrm{class} \end{array}}{\underbrace{\phi_{MBbDd}}}$$

In the class with high fixation probability, for a given value of *q*, the fixation probabilities for the update rules of the class are nearly the same. Let us understand the reason behind this small difference in the fixation probabilities. Recall for the update rules of high fixation probability class both *r* and *t* are not equal to 1, i.e. selection operates at the individual level for the high fixation probability class update rules. We have

D.14 $$\frac{\phi_{\mathrm{wm}}^{N_{1}}(r,t)}{\phi_{\mathrm{wm}}^{N_{1}}(1/r,1/t)}={\left(\frac{r}{t}\right)}^{N_{1}-1}.$$

We first focus on the regime *q* > 1, with *r* = *q*, $t=\frac{1}{q}$,

D.15 $$\frac{\phi_{\mathrm{wm}}^{N_{1}}(r,t)}{\phi_{\mathrm{wm}}^{N_{1}}(1/r,1/t)}=q^{2(N_{1}-1)}\gg 1.$$

As a result, the fraction

D.16 $$\frac{\pi_{\circ }^{j-}}{\pi_{∙}^{j+}}=\frac{1+\frac{M-1-j+t^{\prime }j}{r^{\prime }}\frac{\phi_{\mathrm{wm}}^{N_{1}}(1/r,1/t)}{\phi_{\mathrm{wm}}^{N_{1}}(r,t)}}{1+\frac{r^{\prime }}{t^{\prime }}(M-1-j+t^{\prime }j)\frac{\phi_{\mathrm{wm}}^{N_{1}}(r,t)}{\phi_{\mathrm{wm}}^{N_{1}}(1/r,1/t)}}\ll 1,$$

and consequently, equation (D.2) reduces to

D.17 $$\phi^{⋆}\approx \left(\frac{M-1}{M}\frac{1-\pi_{\circ }^{1-}}{\pi_{∙}^{1+}}+\frac{1}{M}\right)\pi_{∙}^{0+}\phi_{\mathrm{wm}}^{N_{1}}(r,t).$$

In the above equation, $\frac{1-\pi_{\circ }^{1-}}{\pi_{∙}^{1+}}$ can be simplified when *M* is sufficiently small such that $\left(M-2+t^{\prime }\right)$ $\frac{\phi_{\mathrm{wm}}^{N_{1}}\left(\frac{1}{r},\frac{1}{t}\right)}{\phi_{\mathrm{wm}}^{N_{1}}(r,t)}\ll 1$. In this case,

D.18 $$\frac{1-\pi_{\circ }^{1-}}{\pi_{∙}^{1+}}\approx 1\;$$

and

D.19 $$\pi_{∙}^{0+}\approx 1.$$

Hence,

D.20 $$\phi^{⋆}\approx \phi_{\mathrm{wm}}^{N_{1}}(r,t),$$

which is independent of the selection parameters *r*′ and *t*′. This implies that the fixation probability of a mutant on the metastar for an update rule of high class fixation probability (update rule where *r* ≠ 1 and *t* ≠ 1) is the same regardless of the nature of selection at the patch level. This in turn explains why the fixation probability profiles in the high fixation probability class are so similar.

We use similar arguments to study the differences in the fixation probability profiles of the intermediate fixation probability class where either *r* ≠ 1 or *t* ≠ 1. From figure 5, we observe that for a given *q* value, the differences among the fixation probability for different update rules of the class is higher. For the update rules with *r* ≠ 1 and *t* = 1, we have

D.21 $$\frac{\phi_{\mathrm{wm}}^{N_{1}}(r,t)}{\phi_{\mathrm{wm}}^{N_{1}}(1/r,1/t)}=r^{N_{1}-1}.$$

Similarly, for the update rules with *r* = 1 and *t* ≠ 1, we have

D.22 $$\frac{\phi_{\mathrm{wm}}^{N_{1}}(r,t)}{\phi_{\mathrm{wm}}^{N_{1}}(1/r,1/t)}={\left(\frac{1}{t}\right)}^{N_{1}-1}.$$

Substituting *r* = *q* and $t=\frac{1}{q}$, we get,

D.23 $$\frac{\phi_{\mathrm{wm}}^{N_{1}}(r,t)}{\phi_{\mathrm{wm}}^{N_{1}}(1/r,1/t)}=q^{N_{1}-1}.$$

which for *q* > 1 is less than what we have in the class with the high fixation probability (see equation (D.15)). As a result, we expect that in the class with the intermediate fixation probability, the patch level selection is more important.

In the class of low fixation probability with *r* = *t* = 1, since

D.24 $$\frac{\phi_{\mathrm{wm}}^{N_{1}}(r,t)}{\phi_{\mathrm{wm}}^{N_{1}}(1/r,1/t)}=1,$$

the effect of patch selection on the fixation probability is more notable compared with the two other classes. As a result, the differences in the fixation probabilities for the update rules of this class is larger than what we found in the previous two classes. Also, the conditional transition probabilities in equations (D.7) and (D.8) become identical to the transition probabilities of star graph with one individual per node. Therefore, for the case of low fixation probability class update rules, the fixation probability on the metastar starting from a single mutant patch is equivalent to the fixation probability on the star graph starting from a single mutant. The fixation probability of the metastar starting from a single mutant, is then the fixation probability of the mutant on the star graph of size *M* times the probability $\phi_{\mathrm{wm}}^{N_{1}}$.

Let us now focus on the case of *q* < 1. In the class with high fixation probability, for *q* < 1 and $N_{1}\gg 1$,

D.25 $$\frac{\phi_{\mathrm{wm}}^{N_{1}}(r,t)}{\phi_{\mathrm{wm}}^{N_{1}}(1/r,1/t)}=q^{2(N_{1}-1)}\ll 1,$$

and hence,

D.26 $$\frac{\pi_{\circ }^{j-}}{\pi_{∙}^{j+}}=\frac{1+\frac{M-1-j+t^{\prime }j}{r^{\prime }}\frac{\phi_{\mathrm{wm}}^{N_{1}}(1/r,1/t)}{\phi_{\mathrm{wm}}^{N_{1}}(r,t)}}{1+\frac{r^{\prime }}{t^{\prime }}(M-1-j+t^{\prime }j)\frac{\phi_{\mathrm{wm}}^{N_{1}}(r,t)}{\phi_{\mathrm{wm}}^{N_{1}}(1/r,1/t)}}\gg 1.$$

As a consequence, from equation (D.12), we find

D.27 $$\phi^{⋆}\ll 1.$$

Therefore, the effect of patch level selection in the class with high fixation probability is negligible. Similarly, we find that for the class with intermediate fixation probability, $\phi^{⋆}\ll 1$.

Although the variation in fixation probabilities is very small for the high fixation probability class, from figure 5, we find seemingly unintuitive fixation probabilities ordering for the different update mechanisms. Intuitively, an update mechanism with higher selection associated events is expected to yield higher fixation probabilities of beneficial mutants. In our numerical investigation, we observe that this holds only for a finite range of *q*. In fact, for a certain value of *q*, we have

D.28 $$\phi_{\mathrm{MBBdD}}^{⋆}>\phi_{\mathrm{MBBDD}}^{⋆}>\phi_{\mathrm{MbBdD}}^{⋆}>\phi_{\mathrm{MbBDD}}^{⋆},$$

which is counterintuitive. Similarly, based on the numerical calculations, in the class with the intermediate fixation probability, for a range of *q*,

D.29 $$\phi_{\mathrm{MBbdD}}^{⋆}>\phi_{\mathrm{MBbDD}}^{⋆}>\phi_{\mathrm{MbbdD}}^{⋆}>\phi_{\mathrm{MbbDD}}^{⋆},$$

which again is counterintuitive.

In the fixation probability for metastar $\phi^{⋆}$, the selection at the individual level always enters the formula via $\phi_{\mathrm{wm}}^{N_{1}}(r,t)$. Since $\phi_{\mathrm{wm}}^{N_{1}}(\frac{r}{t})=\phi_{\mathrm{wm}}^{N_{1}}(\frac{r}{t})$, we have $\phi_{\mathrm{wm}}^{N_{1}}(r=1,t=1/q)=$ $\phi_{\mathrm{wm}}^{N_{1}}(r=q,t=1)$. Therefore, in the intermediate fixation probability class, two update mechanisms that are similar at the patch level selection, but different at the individual level selection, have the same fixation probabilities. For example, **MBBdd** and **MBbdD**, have the same fixation probabilities. This is the reason that instead of eight, we have four distinct curves for the fixation probabilities of intermediate fixation probability class (see inset of figure 5).

**D.2. Update mechanisms with death first**

A similar analysis using the metastar can be performed for the case of 16 update mechanisms where migration is coupled to death, introduced in table 3. We recover a similar grouping of update mechanisms into three fixation probability classes as encountered for the birth first coupled migration update mechanisms (see figure 6).
